# Osmotic demyelination as a complication of hyponatremia correction: a systematic review

**DOI:** 10.1590/2175-8239-JBN-2022-0114en

**Published:** 2023-07-28

**Authors:** Ananda Pires Bastos, Paulo Novis Rocha

**Affiliations:** 1 Universidade Federal da Bahia, Salvador, BA, Brazil.

**Keywords:** Hyponatremia, Myelinolysis, Central Pontine, Hiponatremia, Mielinólise Central da Ponte

## Abstract

**Background:**

Rapid correction of hyponatremia, especially when severe and chronic, can result in osmotic demyelination. The latest guideline for diagnosis and treatment of hyponatremia (2014) recommends a correction limit of 10 mEq/L/day. Our aim was to summarize published cases of osmotic demyelination to assess the adequacy of this recommendation.

**Method:**

Systematic review of case reports of osmotic demyelination. We included cases confirmed by imaging or pathology exam, in people over 18 years of age, published between 1997 and 2019, in English or Portuguese.

**Results:**

We evaluated 96 cases of osmotic demyelination, 58.3% female, with a mean age of 48.2 ± 12.9 years. Median admission serum sodium was 105 mEq/L and > 90% of patients had severe hyponatremia (<120 mEq/L). Reports of gastrointestinal tract disorders (38.5%), alcoholism (31.3%) and use of diuretics (27%) were common. Correction of hyponatremia was performed mainly with isotonic (46.9%) or hypertonic (33.7%) saline solution. Correction of associated hypokalemia occurred in 18.8%. In 66.6% of cases there was correction of natremia above 10 mEq/L on the first day of hospitalization; the rate was not reported in 22.9% and in only 10.4% was it less than 10 mEq/L/day.

**Conclusion:**

The development of osmotic demyelination was predominant in women under 50 years of age, with severe hyponatremia and rapid correction. In 10.4% of cases, there was demyelination even with correction <10 mEq/L/day. These data reinforce the need for conservative targets for high-risk patients, such as 4–6 mEq/L/day, not exceeding the limit of 8 mEq/L/day.

## RESUMO

**Antecedentes:**

A correção rápida da hiponatremia, principalmente quando grave e crônica, pode resultar em desmielinização osmótica. A última diretriz para diagnóstico e tratamento da hiponatremia (2014) recomenda um limite de correção de 10 mEq/L/dia. Nosso objetivo foi sumarizar os casos publicados de desmielinização osmótica para avaliar a adequação dessa recomendação.

**Método:**

Revisão sistemática de relatos de caso de desmielinização osmótica. Incluímos casos confirmados por imagem ou anatomia patológica, em maiores de 18 anos, publicados entre 1997 e 2019, nas línguas inglesa ou portuguesa.

**Resultados:**

Avaliamos 96 casos de desmielinização osmótica, sendo 58,3% do sexo feminino e com média de idade de 48,2 ± 12,9 anos. A mediana de sódio sérico admissional foi 105 mEq/L e > 90% dos pacientes apresentavam hiponatremia grave (<120 mEq/L). Foram comuns os relatos de distúrbios do trato gastrointestinal (38,5%), etilismo (31,3%) e uso de diuréticos (27%). A correção da hiponatremia foi feita principalmente com solução salina isotônica (46,9%) ou hipertônica (33,7%). Correção de hipocalemia associada ocorreu em 18,8%. Em 66,6% dos casos houve correção da natremia acima de 10 mEq/L no primeiro dia de internamento; a velocidade não foi relatada em 22,9% e em apenas 10,4% foi menor que 10 mEq/L/dia.

**Conclusão:**

O desenvolvimento da desmielinização osmótica foi predominante em mulheres, abaixo de 50 anos, com hiponatremia grave e correção rápida. Em 10,4% dos casos, houve desmielinização mesmo com correção <10 mEq/L/dia. Esses dados reforçam a necessidade de alvos conservadores para pacientes de alto risco, como 4–6 mEq/L/dia, não ultrapassando o limite de 8 mEq/L/dia.

## Introduction

Hyponatremia can be defined as a serum sodium concentration below 135 mEq/L, being the most common electrolyte disturbance in clinical practice. In general terms, it occurs when water intake exceeds the kidney excretion capacity^1^. This ability to excrete water is compromised when there is a reduction in the glomerular filtration rate or when there is an increase in antidiuretic hormone (ADH) levels. In turn, ADH levels can rise in response to appropriate stimuli (reduction in effective intravascular volume or hormone deficiencies) or inappropriate stimuli (certain tumors and medications).

Hyponatremia can be asymptomatic or with neurological manifestations, such as nausea and malaise, headache, disorientation, lethargy, muscle cramps, seizures, coma and respiratory arrest, depending on the severity^1^ and the speed of onset of the disorder^2^.

The symptomatology results from the neuronal adaptive response to extracellular hypotonicity^3^ and may be reversed when sodium correction is performed^4^. Sodium is the main extracellular osmolyte; therefore, acute hyponatremia results in extracellular hypotonicity and the consequent displacement of water into the cells of the central nervous system, causing cerebral edema^4^. As an initial response to this volume gain, neurons transport electrolytes out of cells in order to reduce their osmolarity; chronically, there is also a decrease in intracellular organic osmolytes, such as myoinositol, phosphocreatine/creatine, taurine, glutamine and glutamate. These measures bring intracellular osmolarity closer to extracellular osmolarity and reduce cerebral edema, as well as the occurrence of symptoms and permanent neurological damage^1^.

However, as the restoration of these intracellular organic osmolytes is slow, the rapid correction of chronic hyponatremia leaves the neurons temporarily hypotonic in relation to the extracellular environment, resulting in a sudden loss of water. The abrupt reduction in cell volume causes loss of myelin and oligodendrocytes in the pons and in some extrapontine brain regions, a condition known as osmotic demyelination syndrome or pontine/extrapontine myelinolysis^5^.

Symptoms begin between the second and sixth day after correction of hyponatremia^6^ and include dysarthria, mutism, dysphagia, behavioral and movement disorders, lethargy, confusion, disorientation, obtundation and coma^3^.

It is believed that the development of the condition is related to the severity and duration of hyponatremia, the speed of correction and the patient’s risk factors, such as age, sex, alcoholism, malnutrition and other associated comorbidities^5^. Of these, the correction speed is the most important, as it is the only modifiable factor, as it is under the physician’s control.

Acute hyponatremia, lasting less than 24 hours, seems to have its correction better tolerated to the same sodium level than chronic hyponatremia. There are still uncertainties regarding the safe speed of correcting hyponatremia in order to prevent pontine myelinolysis from occurring, since there are reports of cases in which slow correction also culminated in this pathological state^7,8^. Regardless of whether it is acute or chronic, the recommendation of the European guideline^9^ is that the correction should not exceed 10 mEq/L in the first 24 hours. The North American guideline^10^ recommends a correction limit between 10–12 mEq/L/day in the general population and 8 mEq/L in patients with a higher risk of myelinolysis. He also emphasizes that, in addition to establishing the limit, the daily target correction objective should be 4–8 mEq/L in patients with hyponatremia, and 4–6 mEq/L for those at greater risk of pontine myelinolysis.

Therefore, it is necessary to characterize the cases of myelinolysis published in the literature, investigating the possible causal and predisposing factors associated with its development in patients with hyponatremia, as well as what the current recommendations for the management of this fluid and electrolytic disorder are based on. The European suggestion is based on the analysis of 54 cases of myelinolysis published between 1997 and the publication of the guideline, in 2014. Associated with the divergences in relation to the North American recommendations, it also became the objective of this article to expand the sample in search of updated results.

## Methods

The study is a systematic review of the literature, and it is not necessary to submit the project for approval by the Research Ethics Committee (REC), in accordance with Resolution CNS 466/2012. The study was guided by the PRISMA (Preferred Reporting Items for Systematic Reviews and Meta-Analyses) protocol, with the first step being carried out from the search for descriptors in the MESH (Medical Subject Headings of the U. S. National Library of Medicine).

The keywords “osmotic demyelination”, or “osmotic demyelination”, or “myelinolysis”, or “myelinolysis”, and “pontine” or “pontine”, or “extrapontine”, or “extrapontine, and “hyponatremia” or “hyponatremia” were used for searches in the PubMed/MedLine, Lilacs and Scielo bibliographic research systems.

Original papers involving case reports or series of cases of human beings over 18 years of age, published in English and Portuguese, between January 1997 and December 2019, were included. Only cases of hyponatremia were included, in which the authors reported the values of serum sodium, cases in which there was some intervention to correct hyponatremia and cases with central pontine and/or extrapontine myelinolysis confirmed by imaging tests, such as computed tomography (CT) and magnetic resonance imaging (MRI), or the from postmortem pathological anatomy.

Variables concerning the patients were collected and analyzed, such as sex, age, comorbidities and associated conditions, and medication use. Among the comorbidities, there are the following categories: alcoholism (grouped based on the terms “alcoholic”, “chronic alcohol abuse”, “alcohol abuse”, “alcoholism”, “chronic alcoholism”, “chronic alcoholism”); gastrointestinal or eating disorders (“malnutrition”, “inappetence”, “weight loss”, “malnutrition”, “diarrhea”, “vomiting”, “anorexia”, “gastroenteritis”, “celiac disease”, “eating disorders”, “low food intake”, “gastritis”, “malnutrition”); systemic arterial hypertension; endocrine disorders (such as “hypopituitarism” and “adrenal insufficiency”); potomania (encompassing “high water intake”, “polydipsia”, “psychogenic polydipsia”, “potomania”); infections (combining “pneumonia”, “tuberculosis”, “urinary tract infections”) and psychiatric disorders (including “anxiety”, “major depression”, “schizoaffective disorder”, “psychosis”). Regarding medications, the use of diuretics, antidepressants, anticonvulsants, antipsychotics, benzodiazepines, antidiuretic hormone (ADH) analogues, and non-steroidal anti-inflammatory drugs (NSAIDs) was analyzed.

Data on hyponatremia were also evaluated. The severity of the condition was defined based on the measurement of serum sodium, being considered mild when sodium was between 130 and 134 mEq/L, moderate between 120 and 129 mEq/L, and severe when below 120 mEq/L. When the value was only reported as “less than 100 mEq/L”, we assigned the value 99 mEq/L in our database to enable the calculation of measures of central tendency and dispersion. Regarding management, we have hypertonic saline solution; isotonic saline solution; saline (unreported concentration); water restriction; glucocorticoid; potassium (potassium chloride or phosphate; potassium); or the use of a hypotonic solution in an attempt to reduce sodium when its correction was too quick (hypotonic saline solution, dextrose or glucose solution). As for the correction speed, we sought to identify the variation in sodium in the first 24 hours, being categorized as ≥ 10 mEq/L/day or < 10 mEq/L/day. One paper^11^ reported the value of “approximately 10 mEq/L/day”, having been included in the group ≥ 10 mEq/L/day. Another study^12^ presented the velocity in mEq/L/h; the value was multiplied by 24 to obtain the daily variation.

With regards to the occurrence of myelinolysis, it was separated into simultaneously affecting the pontine and extrapontine regions, exclusively in the center of the pons or only extrapontine.

Categorical variables were summarized using simple and relative frequencies, while continuous variables were summarized using mean ± standard deviation, median, minimum and maximum values. Analyzes were performed using the SPSS statistical package (Statistical Package for the Social Sciences), version 20.

## Results

There were 218 papers in the PubMed/Medline, Lilacs and Scielo databases, equivalent to the search between the period from January 1997 to December 2019.

**Figure 1.**
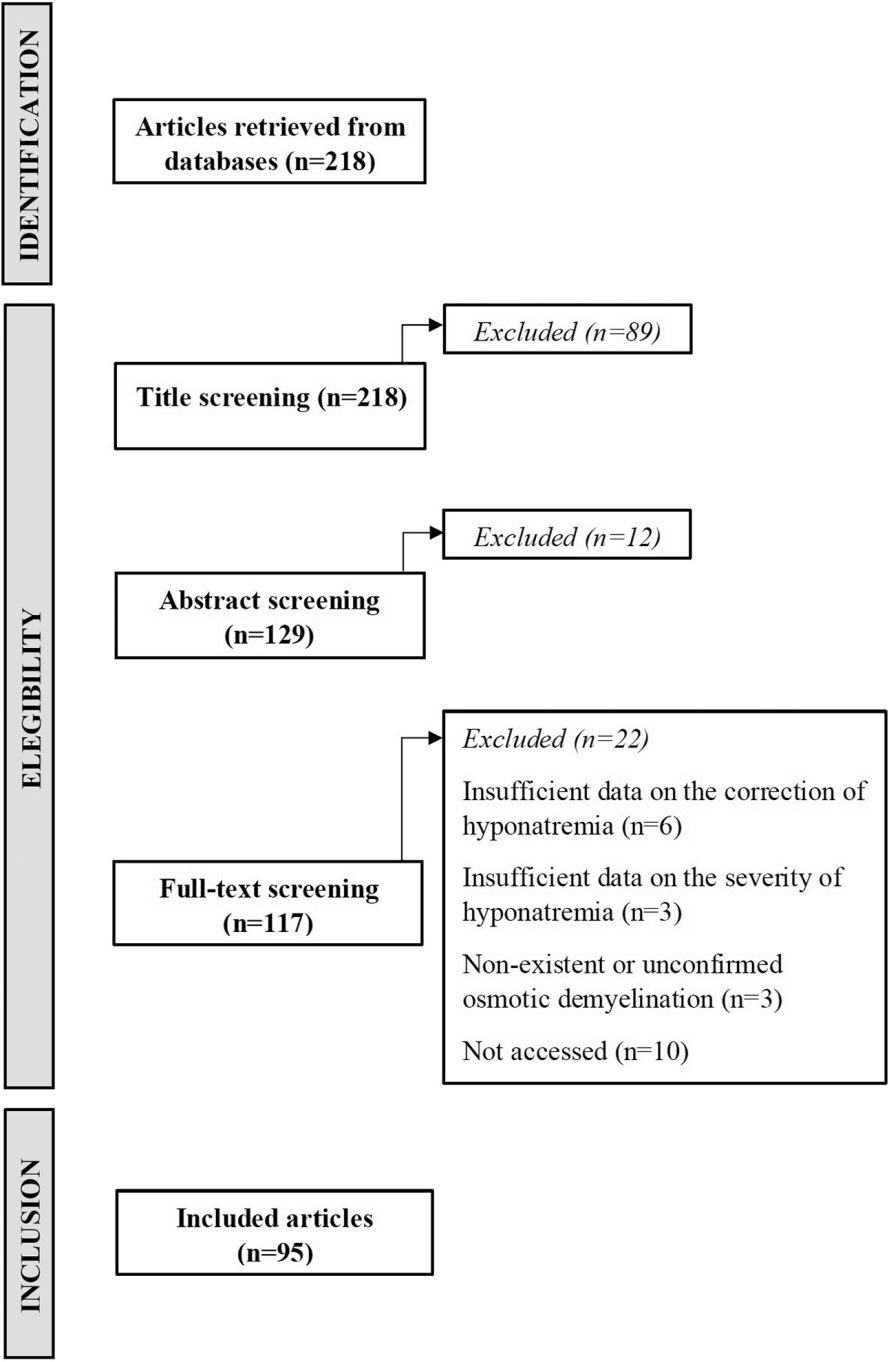
Flowchart describing the process used to select the 95 papers on osmotic demyelination.

After reading the title, 89 papers were excluded, leaving 129 for reading the abstract. After reading the abstract, 12 papers were excluded, leaving 117 for full reading. Unable to access 10 papers. After the complete reading, 12 were excluded and 95 were included to compose the systematic review. This process, from the identification of articles to their inclusion, is described in Figure 1.

Of the 96 cases of myelinolysis evaluated, 56 (58.3%) were female and 40 (41.7%) were male. Age was reported in 94 cases, with a mean of 48.15 ± 12.90 years, a maximum of 76 and a minimum of 19 years. The age groups 19 to 40 years old, 41 to 50 years old and 51 to 60 years old contained 26 cases each (27.7% each), and 16 cases occurred in the age group over 60 years old (17%).

Among the comorbidities of the patients, eating or gastrointestinal tract disorders were reported in 37 cases (38.5%); alcoholism in 30 (31.3%); systemic arterial hypertension in 23 (24%); endocrinological in 16 (16.7%); infections in 13 (13.5%); polydipsia in 10 cases (10.4%); and psychiatric disorders in 10 cases (10.4%).

With regards to the analyzed medications in use, the use of diuretics was reported in 27 cases (28.1%); antidepressants in nine (9.4%); antipsychotics in seven (7.3%); anticonvulsants in four (4.2%); benzodiazepines in six (6.3%); ADH analogue and NSAIDs in three cases each (3.1% each).

**Figure 2.**
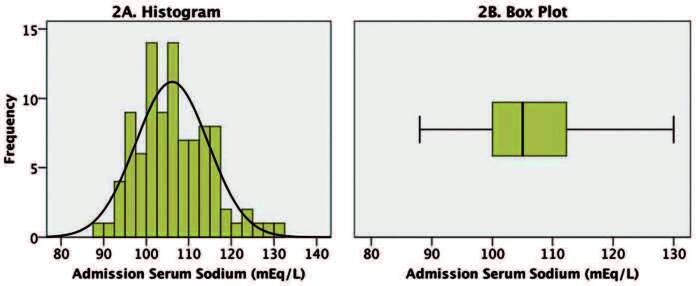
Distribution of admission serum sodium values from 96 patients with osmotic demyelination. A. Histogram with superimposed normal curve; B. Box diagram.

The exact value of serum sodium at admission was reported in 94 of the 95 articles, being approximate in only one of them; in one article, the authors reported that admission sodium was “< 100 meq/L” and we imputed the value of 99 meq/L. The mean was 106 ± 8.5 mEq/L, median 105 mEq/L, maximum 130 and minimum 88 mEq/L (Figure 2AB).

Hyponatremia was classified as severe in 89 cases (93.6%); moderate in five (5.2%); and take it in only one case (1%).

The summary of demographic and clinical data of the included patients, such as gender, age, comorbidities, medications in use, admission sodium and hyponatremia classification, is depicted on Table 1.

**Table 1.** Demographic and clinical data from 96 patients with osmotic demyelination

Variables	N	%
**Age (years)**		
19 to 40	26	27.7
41 to 50	26	27.7
51 to 60	26	27.7
> 60	16	17
**Sex**		
Females	56	58.3
Males	40	41.7
**Comorbidities**		
Food or GIT disorders	37	38.5
Alcoholism	30	31.3
SAH	23	24
Endocrine disorders	16	16.7
Infection	13	13.5
Psychiatric disorders	10	10.4
Potomania	10	10.4
**Medications being used**		
Diuretics	27	28.1
Antidepressant agents	9	9.4
Anti-seizure agents	4	4.2
Anti-psychotic agents	7	7.3
Benzodiazepines	6	6.3
ADH analogue	3	3.1
NSAID	3	3.1
**Hyponatremia classification**		
Mild (130–134 mEq/L)	1	1
Moderate (120–129 mEq/L)	5	5.2
Severe (< 120 mEq/L)	90	93.8
**Diagnostic method**		
MRI	94	97.9
Pathology	2	2.1
**Osmotic demyelination site**		
Pontine and extrapontine	40	41.7
Extrapontine	31	32.3
Pontine	25	26

Hyponatremia management and correction speed are shown in Table 2. Isotonic saline solution was used in 45 cases (46.9%); hypertonic saline solution in 32 cases (33.3%); saline solution of unspecified concentration in six cases (6.3%). Potassium replacement occurred in 18 cases (18.8%); water restriction was reported in only 11 cases (14.7%); use of corticosteroids in six cases (8%); and the use of some hypotonic solution was reported in seven cases (7.3%). It is noteworthy that, in some cases, more than one treatment was instituted.

**Table 2.** Hyponatremia management in 96 patients with osmotic demyelination

Variables	N	%
**Treatment**		
NaCl 0.9%	45	46.9
Hypertonic saline	32	33.3
Saline of non-specified concentration	6	6.3
Fluid restriction	12	12.5
Steroid	7	7.3
KCl	18	18.8
**Na+ correction speed (in 24 hours)**		
Not reported	22	22.9
≥ 10 mEq/L	64	66.6
< 10 mEq/L	10	10.4

Sodium correction in the first 24 hours was reported as greater than or equal to 10 mEq/L in 64 cases (66.6%); less than 10 mEq/L in 10 cases (10.4%); and was not reported in 22 cases (22.9%). Of the papers that presented a correction speed lower than 10 mEq/L in 24 hours, in three there was no specification of the speed, but data present in the text stated that the correction was below this limit. The other articles presented the following velocities: 09 mEq/L/24h; 06 mEq/L/24h; 05 mEq/L/24h; 4.5 mEq/L/24h, 04 mEq/L/24h and 03 mEq/L/24h.

In Table 3, cases of osmotic demyelination were stratified according to correction speed. Patients in the group in which the correction speed was greater than or equal to 10 mEq/L in the first 24 hours had a mean sodium increase of 19.4 ± 6.0 mEq/L against only 5.9 ± 2.1 mEq/ L in the group in which the correction was less than 10 mEq/L (P < 0.001). There was no significant difference between groups for the other evaluated variables.

**Table 3.** Baseline characteristics, therapeutic aspects and osmotic demyelination site stratified by serum sodium correction speed in the first 24 hours

Variables	Serum sodium correction speed in 24 hours		
	Not reported (n = 22)	> or = 10 mEq/L (n = 64)	< 10 mEq/L (n = 10)
Sodium upon admission	107.4 ± 10.4	105.1 ± 7.5	109.4 ± 9.9
Delta sodium 24 h	NR	19.4 ± 6.0	5.9 ± 2.1
Age	51 ± 11	48 ±14	46 ± 12
Females	10 (45.5%)	42 (65.6%)	4 (40.0%)
**Comorbidities**			
GIT disorders	10 (45.5%)	25 (39.1%)	2 (20.0%)
Alcoholism	10 (45.5%)	17 (26.6%)	3 (30.0%)
SAH	7 (31.8%)	14 (21.9%)	2 (20.0%)
Endo disorders	6 (27.3%)	9 (14.1%)	1 (10.0%)
Infection	0 (0.0%)	12 (18.8%)	1 (0.0%)
Psychiatric disorders	4 (18.2%)	6 (9.4%)	0 (0.0%)
Potomania	2 (9.1%)	7 (10.9%)	1 (10.0%)
**Medications**			
Diuretics	6 (27.3%)	19 (29.7%)	2 (20.0%)
Antidepressants	3 (13.6%)	6 (9.4%)	0 (0.0%)
Anticonvulsants	2 (9.1%)	1 (1.6%)	1 (10.0%)
Antipsychotic	3 (13.6%)	4 (6.2%)	0 (0.0%)
Benzodiazepines	2 (9.1%)	4 (6.2%)	0 (0.0%)
ADH analogue	1 (4.5%)	1 (1.6%)	1 (10.0%)
NSAIDs	0 (0.0%)	3 (4.7%)	0 (0.0%)
**Treatment**			
NaCl 0.9%	9 (40.9%)	32 (50.0%)	4 (40.0%)
Hypertonic saline	5 (22.7%)	25 (39.1%)	2 (20.0%)
NS saline conc.	1 (4.5%)	4 (6.2%)	1 (10.0%)
Fluid restriction	3 (13.6%)	7 (10.9%)	2 (20.0%)
Steroid	1 (4.5%)	5 (7.8%)	1 (10.0%)
KCl	4 (18.2%)	11 (17.2%)	3 (30.0%)
Hypertonic solution	0 (0.0%)	7 (10.9%)	0 (0.0%)
**OD site**			
Pontine and Extra P	11 (50.0%)	28 (43.8%)	1 (10.0%)
Extra P	5 (22.7%)	16 (25.0%)	4 (40.0%)
Pontine	6 (27.3%)	20 (31.2%)	5 (50.0%)

Legend: Quantitative variables Age (in years), Admission sodium (in mEq/L) and Delta sodium in 24 h (in mEq/L) were summarized using mean ± standard deviation. Categorical variables were summarized using n (%). Abbreviations: GIT = gastrointestinal tract; SAH = systemic arterial hypertension; Endo = Endocrinological; ADH = antidiuretic hormone; NSAID = Non-steroidal anti-inflammatory drug; Salina conc. NE = Saline of unspecified concentration; Sun. = Solution; OD = Osmotic Demyelination; Extra P = Extra Pontine.

## Discussion

Osmotic demyelination, a rare condition, was, for a long time, difficult to recognize before death, being identified mainly at autopsy. With the advancement of imaging methods, mainly with the advent of CT and MRI, it is now possible to diagnose suspected cases while the patient is still alive. The Clinical Practice Guideline on Diagnosis and Treatment of Hyponatraemia^9^, from 2014, analyzed 54 cases of osmotic demyelination to support its recommendations; in this study, it was possible to expand this number to 96 patients.

Hyponatremia, mainly chronic, is described in the literature as the most important predisposing factor for the development of the complication. However, there were no data regarding the duration of hyponatremia in most of the included studies. This reflects the reality of clinical practice, as when a patient presents to the emergency room with hyponatremia, it is very difficult to establish the moment when this disorder began, which reinforces the importance of designating a safe correction range. Our data show that more than 90% of the patients with demyelination reported in the literature had severe hyponatremia, with a median serum sodium of only 105 mEq/L. This data confirms previous studies^2,9,10,13^ that indicate that the severity of hyponatremia is an important risk factor for the development of osmotic demyelination.

The speed of correction of hyponatremia is considered a crucial point for the development of demyelination. The European guideline^9^ recommends that sodium correction, in any hyponatremic patient, does not exceed 10 mEq/L in the first 24 hours. The North American guideline^10^ distinguishes between patients with hyponatremia who are or are not at increased risk of myelinolysis, as well as a different target and limit for each group. The target refers to the range to be achieved and will guide the correction calculations, being 4–8 mEq/L in patients with habitual risk of hyponatremia, and 4–6 mEq/L for those at greater risk of developing osmotic demyelination. The threshold is defined as a milestone not to be crossed to keep the fix safe. North American recommendations suggest that this should be between 10–12 mEq/L in patients at usual risk and 8 mEq/L in patients at higher risk. Those with serum sodium ≤ 105 mEq/L, hypokalemia, alcoholism, malnutrition and chronic liver disease are classified as the highest risk group.

In this review, we showed that in 64% of the cases of osmotic demyelination there was a correction greater than or equal to 10 mEq/L on the first day of hospitalization; this data was not accurately reported in 22% of cases and was less than 10 mEq/L in only 10.4% of cases. This reinforces the importance of targeting even narrower correction targets so that there is more safety in the treatment of hyponatremia.

Correction of hyponatremia was mainly reported using isotonic (46.9%) or hypertonic (33.7%) saline. The use of some source of potassium was reported in 18.8% of cases. Since the use of potassium accelerates sodium correction, correction of hypokalemia should also be considered a factor for sodium overcorrection and, consequently, greater risk of developing osmotic demyelination. The use of water restriction was reported in only 12.5% of the cases. It is not clear whether this was the percentage of patients who underwent fluid restriction or whether there was underreporting, as many authors may not have recorded this important treatment modality in their reports.

Even with the correction speed of serum sodium greater than or equal to 10 mEq/L in 64 patients, in only seven (10.9%) there was documentation of an attempt to reduce serum sodium using a hypotonic solution. North American recommendations^10^ suggest that, in cases of overcorrection of severe hyponatremia (Na < 120 mEq/L), one may consider reduce serum sodium using desmopressin or 5% glucose solution.

The development of hyponatremia may be associated with the use of medications, and the use of diuretics, especially thiazides, seems to be the most associated with sodium reduction among the analyzed cases, being present in about 28% of them. Medications such as antidepressants, antipsychotics, anticonvulsants, benzodiazepines, ADH analogues and NSAIDs have also been reported in the literature as predisposing to the occurrence of hyponatremia^14,15^. Once the medication that has an effect on natremia is withdrawn, the tendency for sodium to be self-corrected, even without additional interventions for this purpose. This data is important because it is something to be considered in the management of hyponatremic patients.

Likewise, some comorbidities and acute situations can facilitate the development of severe hyponatremia and, consequently, its compensation generates an unexpected increase in sodium. In the cases of osmotic demyelination analyzed here, disorders of the gastrointestinal tract predominated, which include situations with loss of electrolytes and hypovolemia, and others such as anorexia or inappetence, also reported in the literature, are the most prominent among the variables analyzed, comprising 38.5% of the cases. Second, a condition that has been widely described as predisposing to the development of demyelination is alcoholism, with 31.3%. It is noteworthy that, in the only case with mild hyponatremia^16^, which was also properly corrected, the patient was an alcoholic. There are reports of osmotic demyelination without hyponatremia in alcoholics^17^, which may indicate alcoholism as a risk factor for myelinolysis regardless of hyponatremia and/or its correction.

The other conditions evaluated, such as systemic arterial hypertension, endocrinological, fluid or psychiatric disorders and infections, may also be associated in their pathophysiology with alterations in the regulation of natremia (such as syndrome of inappropriate secretion of antidiuretic hormone, SIADH) or through the aforementioned drugs. It is essential to assess which – or what – is the underlying cause of hyponatremia so that it can be properly managed. Reversible situations, such as hypovolemia, should be listed as one more factor to increase sodium, which implies following the defined target speed to avoid overcorrection.

Kallakatta et al.^18^ reported a combined occurrence of pontine and extrapontine myelinolysis as the most common, present in more than 50% of cases, followed by exclusive extrapontine myelinolysis in 28% and central pontine in 20% of cases. In this study, we similarly identified the predominance of pontine and extrapontine in 41.7%, only extrapontine in 32.3% and only pontine in 26%.

The main limitation of this work is related to the lack of uniformity in the case reports, as well as the absence of some relevant information that would enable a better analysis of factors related to the development of the complication, such as the precise speed of hyponatremia correction in 24 hours and the management of fluid and electrolytic disorders.

Adequate sodium correction can be a challenge and, in clinical practice, it is important to pay attention to comorbidities and medications in use that may be related to the development of a chronic disorder, in which a sudden change in natremia is not tolerable, or even when there are manipulable factors and imply hypercorrection if they are not valued. We identified that osmotic demyelination is predominant in younger female patients, who have severe hyponatremia and rapid correction. In 10.4% of cases, even with correction < 10 mEq/L in 24h, there was demyelination. Thus, it is important to identify patients at higher risk and follow more conservative correction recommendations; therefore, we reinforce the North American recommendations for sodium correction. In patients at higher risk, the correction target should be between 4 and 6 mEq/L per day, and should not exceed 8 mEq/L. In other patients, the target should be 4 to 8 mEq/L per day, with a maximum of 10 to 12 mEq/L.
